# Lisfranc Injury: Recent Trends in Management

**DOI:** 10.7759/cureus.43182

**Published:** 2023-08-09

**Authors:** Arnab Sain, Emily Prendergast, Kanishka Wattage, Ahmed Elkilany, Arsany Metry

**Affiliations:** 1 Orthopaedics, Worthing Hospital, University Hospitals Sussex NHS Foundation Trust, Worthing, GBR; 2 Orthopaedics and Trauma, Worthing Hospital, University Hospitals Sussex NHS Foundation Trust, Worthing, GBR

**Keywords:** dorsal bridge plate, arthrodesis, open reduction internal fixation, missed diagnosis, lisfranc injury

## Abstract

Lisfranc injury refers to a group of bony or ligamentous injuries in which one or more of the metatarsals are displaced with respect to the tarsus. These injuries can occur as a result of either high-energy trauma like motor vehicle accidents and falls from height, or low-energy trauma from sports activities. A significant proportion of Lisfranc injuries are missed initially. The effects of delayed and missed diagnosed cases can be devastating as patients may develop progressive midfoot instability, collapse of arch, abduction of forefoot, and post-traumatic osteoarthritis, which can cause chronic pain, stiffness, and foot and ankle complex dysfunction. Favourable outcomes are associated with early diagnosis and prompt treatment. Open reduction and internal fixation (ORIF) with arthrodesis has better results than ORIF alone in functional outcomes. Dorsal bridge plates are currently the preferred mode of fixation due to advantages over trans-articular screws.

## Introduction and background

Lisfranc injuries encompass bony or ligamentous injuries where one or more metatarsal is displaced relative to the tarsus. This term originates from Jaque Lisfrant de Saint-Martin, a French military surgeon and gynaecologist who described both the injury and amputation through the midfoot [1]. Lisfranc injuries can result from high-impact injuries sustained from motorcycle accidents or high velocity falls, or low-impact injuries sustained from sports [2]. Studies report that 20% of Lisfranc injury diagnoses are missed initially, likely due to the intricate anatomy of the midfoot rendering diagnosis and detection of subtle cases difficult using X-ray alone [2-5]. Delayed or missed diagnoses are associated with arch collapse, midfoot instability, post-traumatic osteoarthritis, and forefoot abduction, which cause stiffness, chronic pain, and foot and ankle complex dysfunction [6]. This review will focus on the current literature on Lisfranc injury management.

## Review

Anatomy

The Lisfranc joint complex comprises the first to fifth metatarsals, three cuneiforms, the cuboid, communicating ligaments, capsules, and stabilising tendons. The osseous structure of the midfoot makes it inherently stable. The bases of the metatarsals form an arch-like structure with the second metatarsal acting like a keystone. Between the medial and lateral cuneiform lies the base of the second metatarsal. Studies show that a shallow second tarsometatarsal (TMT) joint mortise increases the risk of Lisfranc injury [7,8].

Ligamentous structures are critical in stabilising the Lisfranc joint and comprise: (i) TMT plantar and dorsal ligaments, which cross every TMT joint. Dorsal displacement in Lisfranc injuries occurs as the dorsal ligaments are weaker; (ii) Inter-metatarsal ligaments, which connect the second to fifth metatarsals; (iii) Lisfranc ligament, the plantar interosseous ligament connecting the medial aspect of the second metatarsal to the lateral aspect of the first cuneiform bone.

The Lisfranc ligament complex encompasses the Lisfranc ligament and the first and second metatarsals’ TMT ligaments. The Lisfranc ligament is the most robust ligament, and the second metatarsal is important in stabilising the midfoot arch [1-3].

Diagnosis

A thorough history and examination are key in assessing Lisfranc injuries. The incidence of missing a Lisfranc injury is 20% and typically occurs in low-energy injuries and polytrauma patients. Missed and delayed diagnoses are associated with devastating long-term disabilities [1-3]. A detailed history of the mode of injury is important including foot position, degree of energy involved, and force direction. Patients typically present in significant pain, unable to weight-bear, with midfoot swelling. The midfoot will be tender on palpation and passive forefoot movement will elicit pain.

Regarding imaging, an X-ray is used as a first-line with weight-bearing anteroposterior, oblique and lateral views. Clinicians should be guided by the clinical picture even if X-rays are normal, as Lisfranc injuries are easily missed. Weight-bearing X-rays and comparisons with the unaffected foot may help in diagnosing subtle injuries. Fluoroscopic stress views are also helpful but they are painful and require anaesthesia [1-3].

On a weight-bearing anteroposterior view of the foot, the borders of the medial cuneiform should align with the borders of the first metatarsal. The medial side of the middle cuneiform should align with the medial side of the second metatarsal. On a weight-bearing oblique view, the medial side of the lateral cuneiform should align with the medial side of the third metatarsal. The medial side of the cuboid should align with the medial side of the fourth metatarsal. On a weight-bearing lateral view, the long axis of the talus should align with the medial and middle foot columns. Disturbance of any of these anatomical relationships indicates Lisfranc injury [1-3].

MRI is useful in detecting minor Lisfranc injuries and for those who are unable to tolerate weight-bearing X-rays. Disturbance of the Lisfranc ligament complex is highly indicative of unstable foot injury [1-3]. CT may be beneficial in assessing minor Lisfranc injuries, particularly for polytrauma patients or those unable to tolerate weight-bearing X-rays, and to demarcate proximal fracture line extension into the navicular, cuboid, or cuneiforms [1-3].

Treatment

Conservative management, i.e. immobilization in a boot, is advised for stable injuries, for example, partial sprains and extra-articular fractures. After four to six weeks, patients can wear normal shoes, weight-bear as tolerated, and begin gentle range-of-movement exercises. Two to three weeks after the injury, weight-bearing X-rays should be repeated to check the injury is not displaced. Although patients’ recovery period is long, most can expect full recovery and minimal long-term complications [1-4].

Unstable bony and ligamentous injuries require operative treatment for better functional outcomes. Any dislocation causing overlying skin or soft tissue tension should be reduced and immobilized using a splint. Short-term external fixation can be used for high-energy injuries when temporary alignment cannot be maintained in a splint. In these cases, definitive fixation surgery is deferred for 10-14 days to allow adequate soft tissue healing.

Open reduction and internal fixation (ORIF) was historically considered the gold-standard treatment for acute Lisfranc injuries whereas arthrodesis/fusion was considered a rescue procedure for post-traumatic osteoarthritis or failed fixation. However, primary arthrodesis is now considered the definitive management in cases with extensive articular damage and a high chance of developing post-traumatic osteoarthritis [9]. It is the choice of treatment for ligamentous Lisfranc injuries as ligamentous injuries have longer healing time than bony injuries and the medial and middle columns of the foot are inessential [1]. ORIF with arthrodesis has a reduced rate of re-operation as hardware removal is less commonly required compared to cases managed by primary ORIF [1,10].

In young athletes, primary ORIF alone is preferred in order to allow the function of the medial column. Arthrodesis for higher-demand athletes may result in extreme stress on surrounding structures, increasing the risk of pseudoarthrosis, metatarsalgia and stress fracture [11].

ORIF can be performed either with trans-articular screws or with a dorsal bridge plate. Previously, Kirschner wires (K-wires) were used for fixation but failure rates were high and therefore used only for the lateral column (fourth and fifth metatarsals). Biomechanical studies demonstrate similar strength of cannulated and cortical screws with distal thread; thus, cannulated screws were the chosen implant for screw fixation as they are easy to insert [12]. However, trans-articular screws cause damage to the articular cartilage.

Dorsal bridge plates do not cause any articular cartilage damage. In one study, dorsal bridge plates had better functional and radiological outcomes compared to trans-articular screws or a combination of screws and plates [13]. However, another study found similar biomechanical outcomes between dorsal bridge plates and trans-articular screws [14].

Misdiagnosed cases are associated with chronic pain, limitation of activity, and post-traumatic arthritis. Chronic Lisfranc injuries are linked to poor outcomes compared to promptly diagnosed and treated cases, likely due to joint malalignment and atypical load distribution prior to treatment. Primary arthrodesis is preferable in these patients to ORIF alone [1]. Primary arthrodesis has shown satisfactory outcomes in most patients in their post-operative functional status [15,16].

## Conclusions

Lisfranc injuries are complex injuries of the foot that can be missed on initial scans. Delayed or missed diagnoses are associated with arch collapse, midfoot instability, post-traumatic osteoarthritis, and forefoot abduction, which cause stiffness, chronic pain, and foot and ankle complex dysfunction. Favourable outcomes are associated with early diagnosis and prompt treatment. ORIF with Arthrodesis has better results than ORIF alone in functional outcomes. Dorsal bridge plates are currently the preferred mode of fixation due to their advantages over trans-articular screws. However, more studies are required to determine the best modality of treatment of these complex injuries.
